# Prevalence, intensity and risk factors of tungiasis in Kilifi County, Kenya: I. Results from a community-based study

**DOI:** 10.1371/journal.pntd.0005925

**Published:** 2017-10-09

**Authors:** Susanne Wiese, Lynne Elson, Felix Reichert, Barbara Mambo, Hermann Feldmeier

**Affiliations:** 1 Institute of Microbiology and Hygiene, Charité University Medicine, Berlin, Germany; 2 WAJIMIDA Jigger Campaign, Dabaso Tujengane CBO, Watamu, Kenya; 3 Department of Pediatrics, Charité University Medicine, Berlin, Germany; 4 Kilifi County Research Group, Kilifi County Hospital, Kilifi, Kenya; Swiss Tropical and Public Health Institute, SWITZERLAND

## Abstract

**Background:**

Tungiasis is a neglected tropical disease caused by female sand fleas (*Tunga penetrans*) embedded in the skin. The disease is associated with important morbidity. Tungiasis is endemic along the Coast of Kenya with a prevalence ranging from 11% to 50% in school-age children. Hitherto, studies on epidemiological characteristics of tungiasis in Africa are scanty.

**Methods:**

In a cross-sectional study 1,086 individuals from 233 households in eight villages located in Kakuyuni and Malanga Sub-locations, Kilifi County, on the Kenyan Coast, were investigated. Study participants were examined systematically and the presence and severity of tungiasis were determined using standard methods. Demographic, socio-economic, environmental and behavioral risk factors of tungiasis were assessed using a structured questionnaire. Data were analyzed using bivariate and multivariate regression analysis.

**Results:**

The overall prevalence of tungiasis was 25.0% (95% CI 22.4–27.5%). Age-specific prevalence followed an S-shaped curve, peaking in the under-15 year old group. In 42.5% of the households at least one individual had tungiasis. 15.1% of patients were severely infected (≥ 30 lesions). In the bivariate analysis no specific animal species was identified as a risk factor for tungiasis. Multivariate analysis showed that the occurrence of tungiasis was related to living in a house with poor construction characteristics, such as mud walls (OR 3.35; 95% CI 1.71–6.58), sleeping directly on the floor (OR 1.68; 95% CI 1.03–2.74), the number of people per sleeping room (OR = 1.77; 95% CI 1.07–2.93) and washing the body without soap (OR = 7.36; 95% CI 3.08–17.62). The odds of having severe tungiasis were high in males (OR 2.29; 95% CI 1.18–44.6) and were very high when only mud puddles were available as a water source and lack of water permitted washing only once a day (OR 25.48 (95% CI 3.50–185.67) and OR 2.23 (95% CI 1.11–4.51), respectively).

**Conclusions:**

The results of this study show that in rural Kenya characteristics of poverty determine the occurrence and the severity of tungiasis. Intra-domiciliary transmission seems to occur regularly.

## Author summary

Tungiasis (sand flea disease) is an ectoparasitic skin disease and belongs to the group of NTDs (Neglected Tropical Diseases). It is caused by sand fleas penetrating into the skin of the feet, causing an inflammatory reaction with pain and itching. Attempts to remove the flea with inappropriate sharp tools are painful and cause bacterial superinfection, eventually leading to restricted mobility. In resource-poor communities without access to health care, prevention is the most valuable control measure. In this study we identified important risk factors for the occurrence of tungiasis and sever disease. The most relevant risk factors were poor hygiene practices and poor housing conditions. Simple control interventions such as having solid walls and floors in the house, improved access to water and washing with soap could reduce the disease burden considerably.

## Introduction

Tungiasis (sand flea disease) is a parasitic skin disease caused by female sand fleas (*Tunga penetrans)* penetrated into the skin of human or animal hosts. Tungiasis belongs to the family of neglected tropical diseases (NTDs) [1,2]. It is prevalent in resource-poor rural communities in **s**ub-Saharan Africa, the Caribbean and South America [3–7]. Children between 5 and 14 years and the elderly bear the highest disease burden with prevalences up to 85% [7]. While the great majority of patients harbours less than 10 embedded sand fleas, single individuals may have hundreds of parasites [8,9]. Once embedded in the skin, typically of the toes, the soles and the heels [10], the flea matures. Within the period of up to five weeks it grows until it reaches the size of a pea, produces and releases eggs and finally dies [11]. Morbidity is related to an intense inflammatory response triggered by the development of sand fleas embedded in the epidermis [10,12,13]. Bacterial superinfection is common and intensifies the inflammation. Inflammation and mutilation of the feet eventually lead to impairment of mobility [12]. Main risk factors found in previous studies in Brazil and Nigeria are poor housing and the presence of animals on the compound [14,15]. Awareness of the public health importance of tungiasis has been growing in Kenya in recent years, but valid data on epidemiological characteristics do not exist. In order to develop a sustainable control program for tungiasis in resource-poor communities along the Kenyan Coast, two population-based studies were performed: one in households and the other in schools. Here, we report the results of the household-based study.

## Materials and methods

### Ethics statement

The study was approved by the Ethics Review Committee at Pwani University, Kilifi County, Kenya; approval number ERC/PhD/010/2014. The custodians and their protégés were informed about the objectives and procedures of the study in their mother language (Giriama or Swahili) by a Community Health Worker (CHW). The right to deny participation and withdraw consent at any given time was clearly explained.

The informed consent form was read out loud word by word in Giriama or Swahili and explained further when required, before any interviews were conducted. Questions of the custodian and the children were discussed and answered by a CHW. Consent was obtained via fingerprint or signature from the legal guardian. The examination was performed in a protected surrounding to guarantee the privacy of the patient. Children and adolescents were only examined in the presence of their caregiver.

Any individuals found to have tungiasis were referred to the local CHWs for treatment and follow up according to their standard protocols which have been approved for use by the Ministry of Health at national and county level. For other illnesses requiring treatment a referral form was prepared by a CHW, and patients were referred to the nearest Health Facility. Washing and treatment were also made available for compound members with tungiasis who did not participate in the study.

The information provided to the households verbally is included as supplementary electronic information along with the consent form which was to be signed (S1 Appendix).

### Study area and study population

The study was performed in eight villages located in Kakuyuni and Malanga Sub-locations of Malindi Sub-county, Kilifi County, eastern Kenya, in the dry season from August to October 2014. In the area tungiasis is endemic with prevalences ranging from 30 to 85% in school age children (S2 Appendix).

In Malindi Sub-county rural communities are small and consist of clusters of two to five houses separated by bush or farm land. The area is divided into two ecological zones: Kakuyuni Sub-location, a very densely populated area in the coastal strip with homesteads located side by side. It has a tropical climate with an average annual rainfall of 1,200 mm, temperatures ranging from 28–34°C and high humidity most of the year. Malanga Sub-location is located inland and is much drier with average annual rainfall of 400 mm. Homesteads are located about 100 m from each other in this area. There are two rainy seasons: one between March and May and the other between October and November, interspersed with dry seasons.

Malindi Sub-county has a population of 272,000 with 42.3% being under 15 years of age. The population included in the survey are entirely of the Giriama tribe. While 55% of households have access to piped water and 60% to improved sanitation, only 17% have access to electricity (Malindi Public Health Office 2015). Many of the people live in mud-walled houses with a thatch roof and sandy floor (First Kilifi County Integrated Development Plan 2013–2017). For Kilifi County as whole the poverty rate (i.e. < 1 US$ per day) is 71.4% (http://www.crakenya.org/county/kilifi/). The majority of the population in the study area practice subsistence agriculture, charcoal burning and small scale businesses. The main foodstuffs cultivated are maize, cassava, coconuts, and mangoes.

### Study design

The study was a cross-sectional survey of a random sample of households in Kakuyuni and Malanga Sub-locations, Kilifi County, Kenya. These sublocations were selected because no intervention against tungiasis had been performed so far.

For this study a household was classified as a single structure/house. Since most people live in homesteads of extended families, sharing eating, washing and sanitation facilities, we selected one structure/house per homestead in a standardized manner, always choosing the first house on the left when entering the compound.

Individuals of any age and sex were eligible for participation as long as they had spent at least 4 nights per week in the selected household for the last three months. To be included, a household needed to have someone over the age of 18 present at the time of the visit to sign the consent forms and respond to the interview questions.

During the preparation phase contact was made with the County and District leadership in both the Ministry of Health and the Ministry of Education, the Zonal Education Officer and the Community Health Officers to obtain their approvals and support for the study. We held meetings with all CHWs in each **S**ub-location, gave specific training on tungiasis and explained the aims and procedures of the study, emphasizing that participation was completely voluntary and subjects had the opportunity to withdraw from the study at any point of time.

The study was carried out between August 13 and October 5, 2014, i. e. during a dry season. A total of 1,086 individuals from 233 households in eight villages were included in the study.

Data were collected through a door-to-door survey of the selected households with the help of local CHWs. Eligible patients were explained the procedure and were asked for consent. In case of minors a caregiver (usually the mother) was asked to provide informed consent. If household members were not present during our first visit, we returned to the house on one further occasion. Individuals who could not be reached at home during the second visit were invited to come to the local health facility within the next days. Household members who could not be examined on any occasion were not included in the study.

In order to identify risk factors for the occurrence of tungiasis and severe disease, we requested information about demographic, socio-economic, environmental and behavioural characteristics of the individuals and the household. Structured interviews were conducted with the head of household (usually the mother) using a pre-tested questionnaire in Giriama or Swahili. Environmental, socioeconomic and some behavioural risk factors were assessed at the household level, other risk factors were assessed on the individual level.

Since cash flow does not correctly indicate the economic status of a household in low-income communities [16,17], we used an asset score similar to the one previously established for cutaneous larva migrans, another neglected tropical skin disease associated with poverty [18]. The score is composed of the following assets:

Presence in the household of a radio (2 points), television (5 points), fridge (5 points), gas/solar lamp (1 point); possession of at least one mobile phone (1 point), bicycle (3 points) and motor bike (10 points). The score can vary between 0 and 27 points.

For the diagnosis of tungiasis, the feet of the patients were carefully washed with soap in a basin. Each individual was examined for tungiasis based on a standardized procedure [3]. Since a high number of lesions at the feet frequently coincides with the presence of ectopic lesions at the hands [19], we also systematically examined the hands of the patients. Patients were also asked whether they had tungiasis lesions in other regions of the body. Lesions were staged according to the Fortaleza classification and counted [11]:

- stage I: penetrating sand flea
- stage II: brownish/black dot with a diameter of 1–2 mm surrounded or not by an erythema
- stage III: circular yellow-white watch glass-like patch with a diameter of 3–10 mm and with a central black dot
- stage IV: brownish-black crust with or without surrounding necrosis

Stage I to III are viable sand fleas; in stage IV the parasite is dying or already dead [11] Lesions manipulated with a sharp instrument (by the patient or their caregiver) with the intention to remove the embedded parasite were documented as manipulated lesions. Based on the number of lesions present, the intensity of tungiasis was classified as light (1–5 lesions), moderate (6–30 lesions) or high (>30 lesions) [14].

### Statistical analysis

The data were entered into an Excel database (Excel Version 2013, Microsoft, Redmont, Washington, USA), checked for errors which might have occurred during data entry and then transferred to SPSS (PASW Statistics 18.0, SPSS Inc., Chicago, IL, USA). The data analysis was carried out using the Analysis ToolPack Add-In (Microsoft, Redmont, Washington, USA). Graphs were created with the PowerPivot Add-In (Microsoft, Redmont, Washington, USA). Relative frequencies were compared with the Chi-square test and Fisher’s exact test. The Spearman rank correlation coefficient was calculated to determine the significance of correlations. Odds ratios together with their 95% confidence interval (CI) were calculated first in a bivariate analysis. In a second step, variables which were significantly (p < 0.05) related to the occurrence of tungiasis and/or severe disease were entered in a multivariate logistic regression model with stepwise forward inclusion of variables to identify independent exposure variables. Factors which showed up as significant in the bivariate analysis but were assessed only in individuals older than 18 were not included in the logistic regression model. For risk factors suitable for an intervention, population attributable fractions (PAF) were calculated. The PAF, calculated as % exposed among cases x attributable risk (AR), is the fraction of cases which would not have occurred if an exposure had been avoided, assuming the exposure is causal and the other risk factors in the population remain unchanged. AR is calculated as (OR– 1)/OR and is the risk of tungiasis in the exposed group due to the exposure. The sample size of this study was estimated based on field studies performed in Brazil and Nigeria and contained the following assumptions: control-case-ratio 1:3; hypothetical proportion of controls with exposure 30%; least detectable odds ratio 1.75; power of the test 0.90; confidence level 0.95. This would require 205 cases and 610 controls. To account for uncertainties and drop out we attempted to include a sample of 1000 individuals.

## Results

### Characteristics of the study population

**Fig 1:**
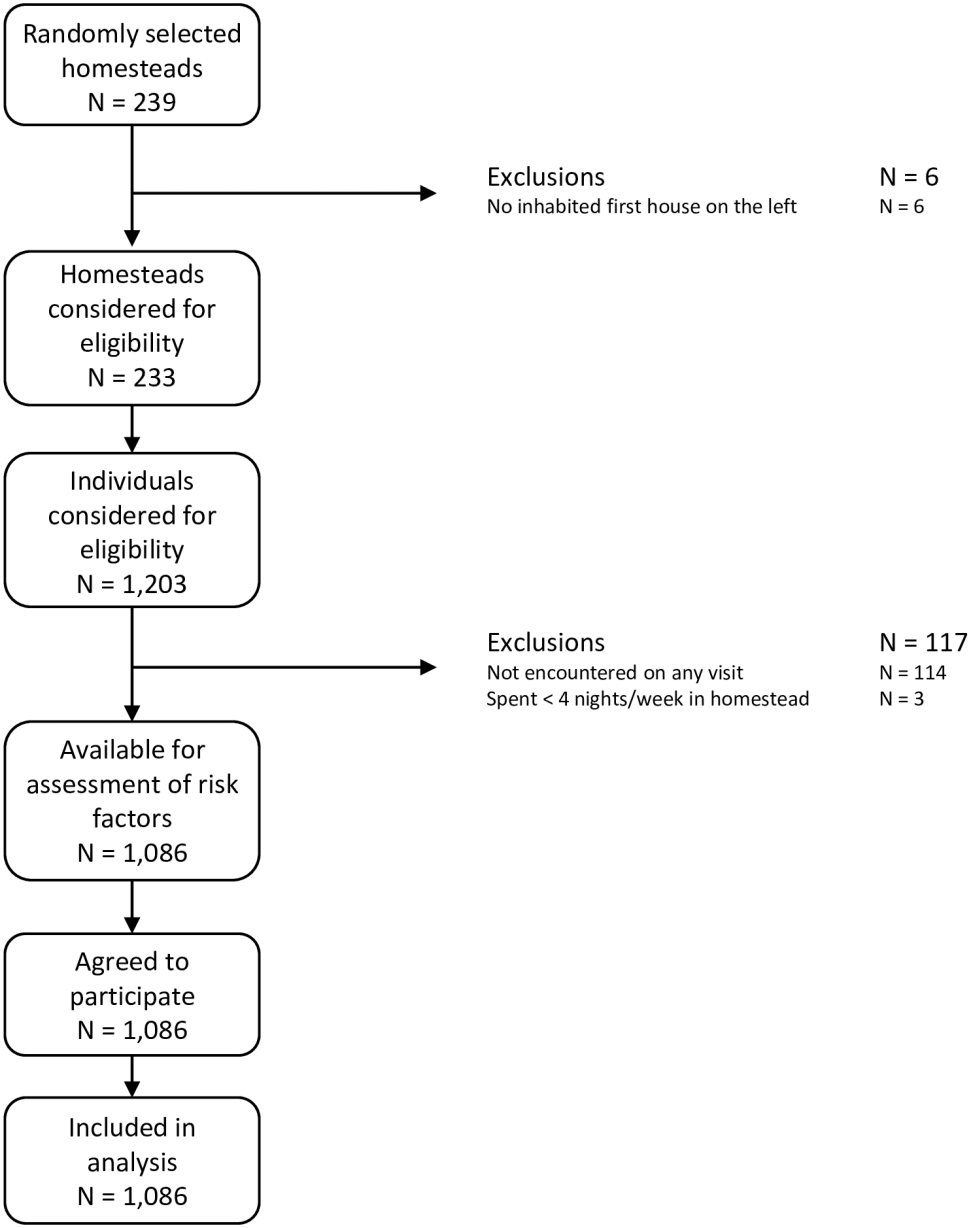
Flow chart of study population.

Of the 239 homesteads visited 233 fulfilled the criterion of having an inhabited first house on the left. Of the 1,203 individuals living in these households, 114 (72 males and 42 females) were not encountered on any of the visits, reducing the study population to 1,089. Of these, three did not fulfil the inclusion criterion of having spent at least four nights per week in the selected homestead during the last three months. Thus, the number of individuals available for the assessment of risk factors was 1,086, all of which agreed to being interviewed and examined (Fig 1). Three hundred and twenty four patients (70 households) were recruited from Kakuyuni, 221 (41) from Goshi, and 172 (43) from Vihingoni community in Kakuyuni Sub-location; 116 (24) from Mtoroni, 27 (5) from Yembe, 133 (28) from Kadzitsoni, 76 (18) from Chembe and 17 (4) from Bahati community in Malanga Sub-Location.

The study population comprised 57.3% females, and 58.6% under the age of 15 years. Of those over 18 years, 54.1% reported being Christians while 19.6% were Muslims, 31.4% were illiterate and a further 34% had not completed primary school education. The majority of houses (89%) had dirt floors and mud walls (84.5%), did not have improved latrines (56.7% used the bush, 32.6% used traditional latrines) and shared community taps for their source of water (83.7%) (Tables 1 and 2).

**Table 1:** Demographic characteristics of the study population (n = 1,086 individuals).

Characteristic	Frequency	(%)
Sex		
Female	622	57.3
Male	464	42.7
Age (years)		
0–4	211	19.4
5–9	245	22.6
10–14	180	16.6
15–19	80	7.4
20–39	189	17.4
40–59	114	10.5
≥ 60	64	5.9
n.k.	3	0.3
Village		
Kakuyuni	324	29.8
Goshi	221	20.3
Vihingoni	172	15.8
Mtoroni	116	10.7
Yembe	27	2.5
Kadzitsoni	133	12.2
Chembe	76	7.0
Bahati	17	1.6
Religion^a^		
None	19	4.9
Muslim	76	19.6
Christian	210	54.1
Traditionist	76	19.6
n.k.	7	1.8
Education^a^		
Illiterate	122	31.4
Primary school not completed	132	34.0
Primary school completed	133	34.3
n.k.	1	0.3
Occupation^a^		
Unemployed	89	22.9
Farmer	179	46.1
Other occupation	113	29.1
n.k.	7	1.8

^a^ information on religion, education and occupation were only collected for adults ≥ 18 years (n = 381)

n.k. = not known

**Table 2:** Socio-economic characteristics of the study population (n = 233 households).

Characteristic	Frequency	(%)
Housing		
Type of floor material		
Cement/stone	26	11.2
Smeared mud	136	58.4
Sand/dust	70	30.0
Mixed mud and sand	1	0.4
Type of wall material		
Stone	31	13.3
Mud	197	84.5
Mixed stone and mud	5	2.1
Type of roof material		
Makuti^a^	112	48.1
Mabati^b^	118	50.6
Mixed makuti and mabati	1	0.4
Tiles	2	0.9
Sanitation		
Toilet		
Flush toilet	10	4.3
Ventilated pit latrine	15	6.4
Traditional latrine	76	32.6
Bush	132	56.7
Waste disposal		
Pit	85	36.5
Pile	100	42.9
Spread	47	20.2
Compost	1	0.4
Water source		
Tap on compound	36	15.5
Shared community tap	195	83.7
Mud puddles	2	0.9
Time to reach water source (min)		
0–4	73	31.3
5–9	53	22.7
10–14	42	18.0
15–19	20	8.6
20–29	12	5.2
≥ 30	33	14.2
Healthcare		
Time to reach next health facility (min)		
0–9	16	6.9
10–19	40	17.2
20–29	43	18.5
30–39	70	30.0
40–49	16	6.9
50–59	2	0.9
≥ 60	46	19.7
Economic status		
Monthly income per household (KSh)^c^		
0–4850	87	37.3
> 4850	40	17.2
n.k.	106	45.5
Number of meals per day		
1	6	2.6
2	62	26.6
> 2	165	70.8
Land ownership		
Own	228	97.9
Rent	3	1.3
Squatt	2	0.9
Domestic animals		
Animals on compound		
Any animal	205	88.0
Dogs	59	25.3
Cats	59	25.3
Goats	140	60.1
Cows	70	30.0
Chicken	172	73.8
Ducks	42	18.0

^a^ palm leaves

^b^ corrugated iron sheets

^c^ KSh 4850 correspond to the minimum wage in Kenya for an unskilled worker in agricultural industry at the time of the survey and is equivalent to ~ 55 USD

n.k. = not known

**Fig 2:**
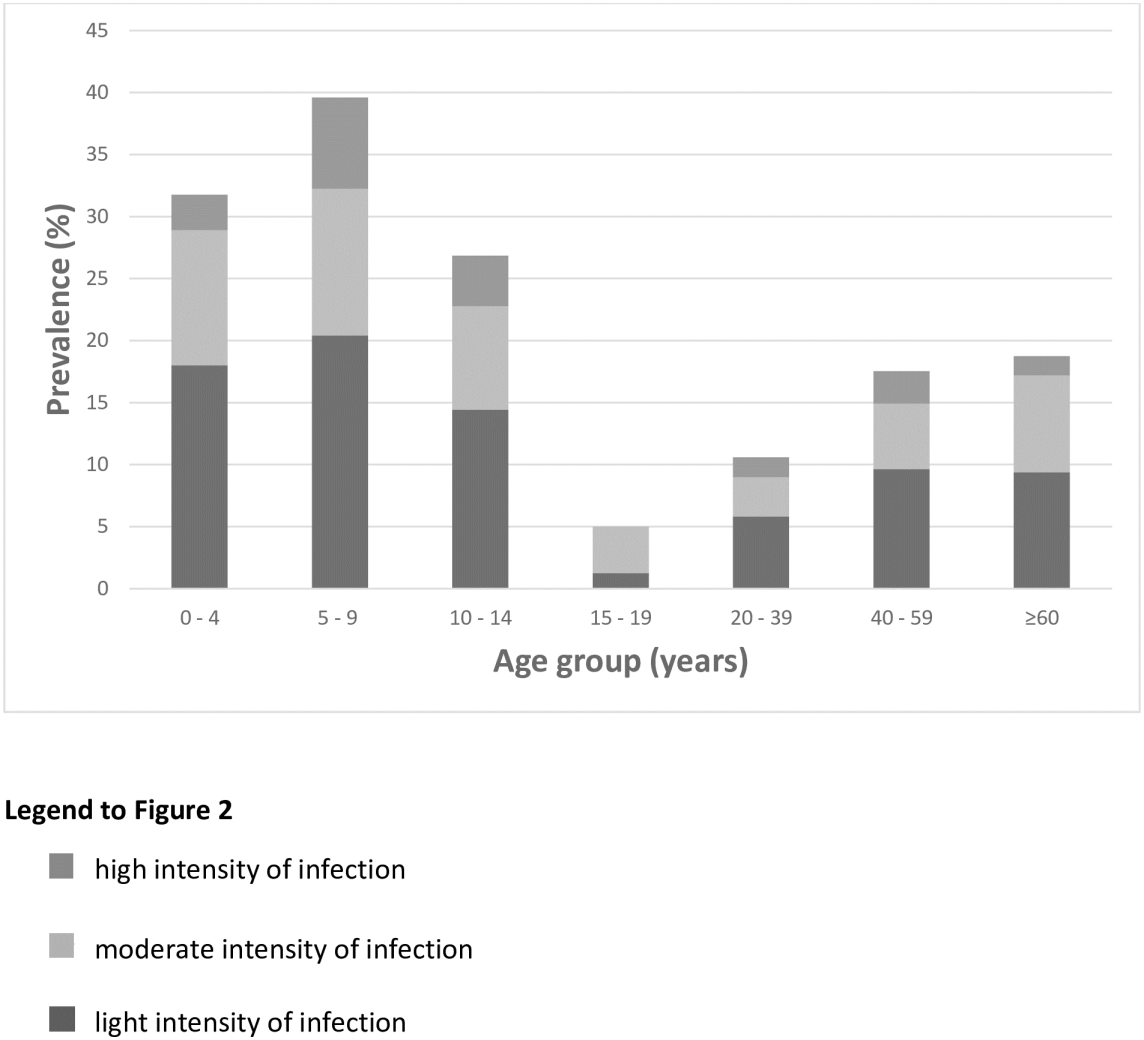
Age-specific prevalence and intensity of tungiasis in the study area. Column heights indicate overall prevalence in age groups.

**Fig 3:**
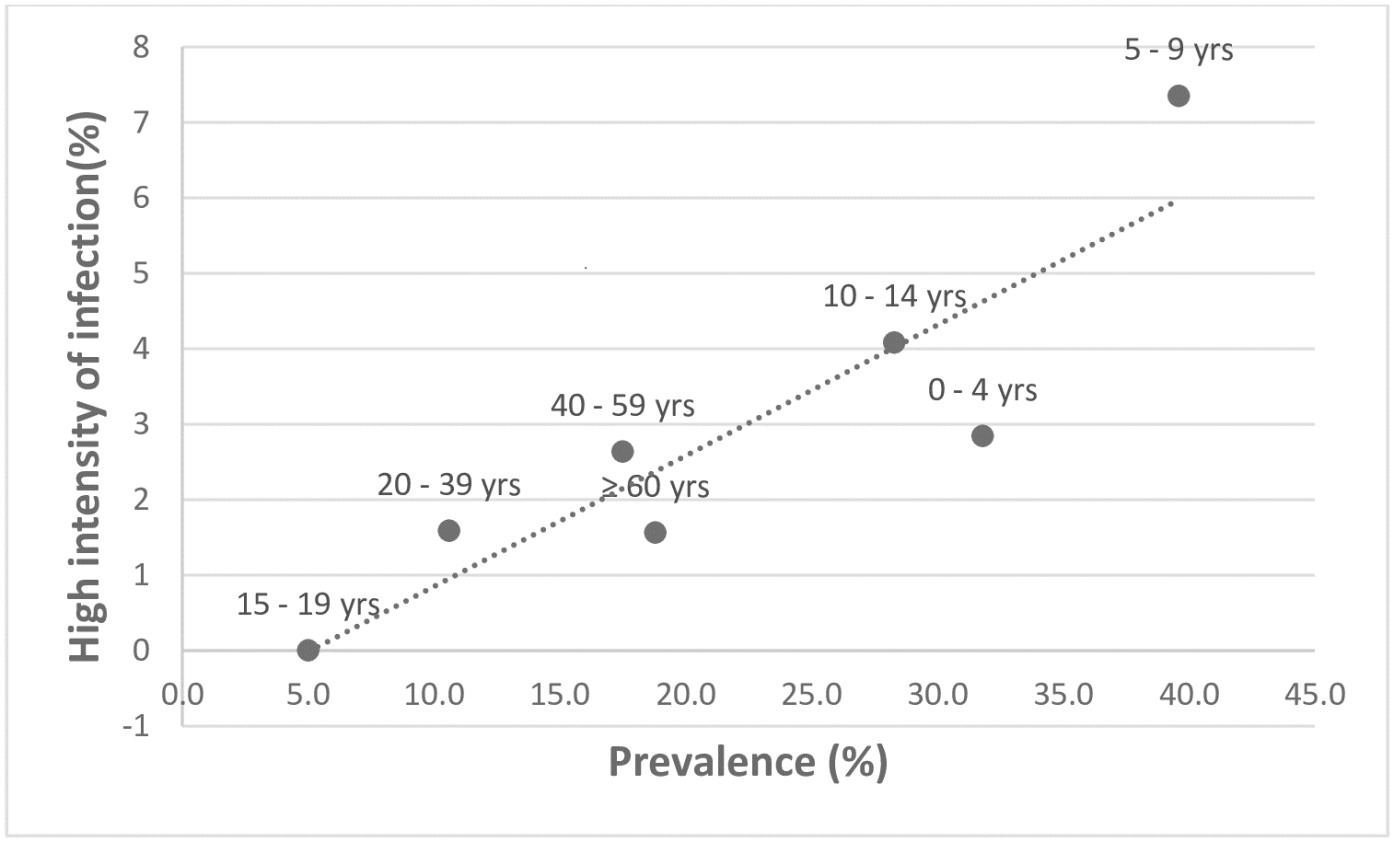
Correlation between age-specific prevalence and age-specific frequency of high intensity of infection (> 30 lesions); rho = 0.90, p = 0.006.

The overall prevalence of tungiasis in the study population was 25.0% (95% CI 22.4–27.5%), but in 42.5% of the households at least one individual had tungiasis. Of those with tungiasis, 52.8% had a light (1 to 5 lesions), 32.1% a moderate (6 to 30 lesions) and 15.1% a high intensity of infection (>30 lesions). Five percent of the patients had ectopic lesions, almost exclusively on the hands. Age-specific prevalences and intensity of infection are shown in Fig 2. There was a tendency of higher occurrence of tungiasis in elderly individuals living alone, although it was not significant (p = 0.2111). In 14 single-person households there were two adults < 40 years without tungiasis, six 40 to 59 year olds of whom 2 had tungiasis and six > 60 year olds of whom 4 had a mild to severe tungiasis. The prevalence of infection and high intensity of infection correlated significantly (Fig 3) (rho = 0.90, p = 0.0059), with the highest prevalence being in the under 15 year olds and over 40 years. The youngest patient was four months old, 4 patients were younger than one year, while the oldest patient was 80 years old.

### Risk factor analysis

Prevalence and severity of tungiasis varied considerably between the villages with Yembe and Bahati having a prevalence of 59.3% and 64.7% respectively, while Mtoroni and Vihingoni had prevalences of 7.8% and 13.4% (Table 3). Residence in Yembe and Bahati was a significant risk factor for tungiasis (OR 17.3 and 21.8 respectively, p<0.0001) and in Kakuyuni for both occurrence of tungiasis (OR 6.5, p<0001) and severe tungiasis (OR 9.2, p<0.05). Tables 3 to 5 show demographic, socio-economic, behavioral, environmental and geographic risk factors in the bivariate analysis.

**Table 3:** Bivariate analysis of geographic risk factors (n = 1,086 individuals).

Exposure variable		n	Frequency of tungiasis		Presence of tungiasis		Presence of severe Tungiasis (> 30 lesions)	
			(%) any	(%) heavy	OR (95% CI)	P value	OR (95% CI)	P value
**Location**								
**Malanga Sublocation**^a^	Mtoroni	116	7.8	0.9	Reference			
	Yembe	27	59.3	3.7	17.29 (6.20–48.23)	**<0.0001**	4.42 (0.27–73.05)	0.2987
	Kadzitsoni	133	33.8	3.8	6.08 (2.82–13.12)	**<0.0001**	4.49 (0.52–30.02)	0.1732
	Chembe	76	10.5	0.0	1.40 (0.51–3.80)	0.5106	0.50 (0.02–12.52)	0.6754
	Bahati	17	64.7	0.0	21.80 (6.53–72.74)	**<0.0001**	2.20 (0.09–56.17)	0.6334
**Kakuyuni Sublocation**	Kakuyuni	324	35.5	7.4	6.54 (3.19–13.40)	**<0.0001**	9.20 (1.23–68.80)	**0.0306**
	Goshi	221	19.9	2.7	2.96 (1.39–6.30)	**0.0050**	3.21 (0.38–26.98)	0.2831
	Vihingoni	172	13.4	2.3	1.84 (0.82–4.12)	0.1416	2.74 (0.30–24.81)	0.3704

^a^ The village with the lowest prevalence was used as reference.

**Table 4:** Bivariate analysis of demographic, housing, economic and behavioral risk factors (n = 1,086).

Exposure variable		n	Frequency of tungiasis		Presence of tungiasis^a^		Presence of severe tungiasis (> 30 lesions)	
			(%)any	(%)heavy	OR (95% CI)	P value	OR (95% CI)	P value
Demographic characteristics								
Sex	Female	622	21.2	2.4	Reference			
	Male	464	30.0	5.6	1.59 (1.20–2.09)	**0.001**	2.40 (1.26–4.59)	**0.008**
Age group (years)	0–4	211	31.8	2.8	8.84 (3.10–25.17)	**<0.001**	5.09 (0.28–91.45)	0.27
	5–9	245	39.6	7.3	12.45 (4.41–35.15)	**<0.001**	13.09 (0.78–219.77)	0.07
	10–14	180	28.3	5.6	7.51 (2.61–21.60)	**<0.001**	9.92 (0.57–171.31)	0.11
	15–19	80	5.0	0.0	Reference			
	20–39	189	10.6	1.6	2.25 (0.74–6.80)	0.15	3.02 (0.15–59.17)	0.47
	40–59	114	17.5	2.6	4.04 (1.33–12.33)	**0.01**	5.05 (0.26–99.21)	0.29
	≥60	64	18.8	1.6	4.38 (1.34–14.35)	**0.01**	3.80 (0.15–94.95)	0.42
Persons per household	1–3	148	18.2	4.7	Reference			
	4–6	453	27.4	4.2	1.69 (1.06–2.69)	**0.03**	0.88 (0.36–2.14)	0.78
	≥7	485	24.7	3.1	1.47 (0.93–2.35)	**0.10**	0.64 (0.26–1.61)	0.34
Children/household	0–3	454	23.6	2.9	Reference			
	4–5	324	24.4	4.9	1.05 (0.75–1.46)	0.79	1.76 (0.84–3.72)	0.13
	≥6	308	27.6	3.9	1.24 (0.89–1.72)	0.21	1.38 (0.62–3.06)	0.43
Adults/household	0–1	224	29.9	6.3	1.65 (0.89–3.07)	0.11	10.81 (0.64–183.47)	0.09
	2–3	784	24.0	3.4	1.22 (0.69–2.17)	0.49	5.70 (0.34–94.35)	0.22
	≥4	78	20.5	0.0	Reference			
Housing								
Type of floor material	Cement/stone	129	11.6	0.0	Reference			
	Smeared mud	661	22.7	4.2	2.23 (1.26–3.94)	**0.005**	11.65 (0.71–192.08)	0.08
	Sand/dust	293	36.2	4.4	4.31 (2.39–7.76)	**<0.0001**	12.47 (0.74–211.31)	0.08
Type of wall material	Stone	153	8.5	0.0	Reference			
	Mud	901	27.6	4.6	4.11 (2.29–7.40)	**<0.0001**	14.81 (0.91–241.97)	0.06
	Mixed	32	28.1	0.0	4.21 (1.62–10.98)	**0.003**	4.72 (0.09–242.42)	0.43
Type of roof material	Mabati	553	21.9	1.4	Reference			
	Makuti	519	28.7	6.4	1.44 (1.09–1.90)	**0.01**	4.36 (2.12–10.11)	**0.0001**
	Both	7	0.0	0.0	0.24 (0.01–4.18)	0.32	4.38 (0.23–81.08)	0.33
	Other	7	14.3	0.0	0.60 (0.07–4.99)	0.63	4.38 (0.23–81.08)	0.33
Location of kitchen	Outside the house	757	25.8	3.6	Reference			
	Inside the house	329	23.1	4.3	0.87 (0.64–1.17)	0.35	1.20 (0.62–2.32)	0.58
Number of sleeping rooms	≥4	68	13.2	0.0	Reference			
	3	179	20.7	2.8	1.71 (0.78–3.76)	0.18	4.32 (0.24–79.15)	0.32
	2	435	23.2	2.3	1.98 (0.95–4.14)	0.07	3.38 (0.20–58.36)	0.40
	1	404	30.7	6.4	2.90 (1.40–6.04)	**0.004**	9.59 (0.58–159.27)	0.11
Persons/sleeping room	<3	403	18.4	2.2	Reference			
	3–4	366	24.3	1.9	1.43 (1.01–2.02)	**0.04**	0.85 (0.31–2.32)	0.75
	4,5–6	195	33.8	8.7	2.27 (1.54–3.36)	**<0.0001**	4.18 (1.83–9.56)	**<0.001**
	≥7	122	34.4	6.6	2.33 (1.49–3.66)	**<0.001**	3.07 (1.16–8.14)	**0.02**
Sleeping situation of children	Raised bed^a^	910	23.2	2.9	Reference			
	Floor	146	36.3	8.2	1.89 (1.30–2.74)	**<0.001**	3.04 (1.50–6.18)	**0.002**
	Taking turns	30	23.3	10.0	1.01 (0.43–2.38)	0.98	3.78 (1.08–13.25)	**0.04**
Sanitation								
Water source	Tap on compound	159	22.0	3.8	Reference			
	Shared community tap	918	24.9	3.6	1.18 (0.79–1.76)	0.42	0.95 (0.39–2.31)	0.91
	Mud puddles	9	77.8	22.2	12.40 (2.46–62.39)	**0.002**	7.29 (1.24–42.80)	**0.003**
Toilet	Flush toilet	36	5.6	0.0	Reference			
	Ventilated pit latrine	88	17.0	0.0	3.49 (0.76–16.14)	0.11	0.41 (0.01–21.18)	0.65
	Traditional latrine	314	25.8	4.8	5.91 (1.39–25.15)	**0.01**	3.78 (0.22–64.48)	0.35
	Bush	648	26.7	4.0	6.19 (1.47–26.05)	**0.01**	3.11 (0.19–52.02)	0.43
Waste disposal	Pit	415	24.1	1.9	Reference			
	Pile	465	23.7	4.7	0.98 (0.72–1.33)	0.87	2.53 (1.11–5.74)	**0.03**
	Spread	199	30.7	5.5	1.39 (0.96–2.03)	0.08	2.98 (1.18–7.52)	**0.03**
	Compost	7	0.0	0.0	0.21 (0.01–3.70)	0.28	3.20 (0.19–60.59)	0.43
Time to reach water source (min)	0–4	341	25.2	4.4	Reference			
	5–9	272	29.8	5.5	1.26 (0.88–1.80)	0.21	1.27 (0.61–2.64)	0.53
	10–14	202	21.8	3.0	0.83 (0.55–1.25)	0.36	0.67 (0.25–1.74)	0.41
	15–19	79	19.0	0.0	0.69 (0.38–1.28)	0.24	0.13 (0.01–2.24)	0.16
	20–29	46	23.9	8.7	0.93 (0.45–1.92)	0.85	2.07 (0.66–6.53)	0.21
	≥30	146	23.3	0.7	0.90 (0.57–1.42)	0.65	0.15 (0.02–1.15)	0.07
Frequency of washing	Twice a day	828	21.7	3.0	Reference			
	Once a day	236	35.6	6.4	1.99 (1.45–2.72)	**<0.0001**	2.18 (1.13–4.21)	**0.02**
	Less often	22	31.8	4.5	1.68 (0.67–4.18)	0.27	1.53 (0.20–11.82)	0.68
Use of soap	Always	566	22.8	3.2	Reference			
	Sometimes	486	25.5	4.3	1.16 (0.87–1.54)	0.30	1.37 (0.72–2.61)	0.33
	Never	34	52.9	5.9	3.81 (1.89–7.69)	**<0.001**	1.90 (0.42–8.56)	0.40
	Always	566	22.8	3.2				
Economic status								
Income per capita (KSh/month)^b^	>3400	25	12.0	0.0	Reference			
	1000–3400	166	18.1	1.2	1.62 (0.45–5.76)	0.46	0.78 (0.04–16.61)	0.87
	<1000	414	27.1	2.9	2.72 (0.80–9.26)	0.11	1.58 (0.09–27.52)	0.75
	n.k.	481	26.2	5.6	2.60 (0.77–8.85)	0.13	3.09 (0.18–52.04)	0.43
Asset score	0–4	761	27.2	4.3	7.85 (1.05–58.71)	**0.04**	2.07 (0.12–34.85)	0.61
	5–15	303	20.8	2.6	5.51 (0.73–41.77)	0.10	1.29 (0.07–23.16)	0.86
	≥16	22	4.5	0.0	Reference			
Number of meals/day	>2	773	23.7	3.5	Reference			
	2	291	27.8	4.1	1.24 (0.92–1.69)	0.16	1.19 (0.59–2.38)	0.63
	1	22	31.8	9.1	1.50 (0.60–3.75)	0.38	2.76 (0.61–12.43)	0.19

^a^ Bed height was not assessed systematically, but was approximately 45 cm above the ground (personal observation)

^b^ KSh 4850 correspond to the minimum wage in Kenya for an unskilled worker in agricultural industry at the time of the survey and is equivalent to ~ 55 USD

n.k. = not known

**Table 5:** Bivariate analysis of educational, occupational and environmental risk factors (n = 1,086).

Exposure variables		n	Frequency of tungiasis		Presence of tungiasis		Presence of severe tungiasis (> 30 lesions)	
			% (any)	(%) heavy	OR (95% CI)	p-value	OR (95% CI)	p-value
**Education**^a^	Primary school completed	133	6.8	0.8	Reference			
	Primary school not completed	132	15.9	3.8	2.61 (1.15–5.93)	**0.02**	5.20 (0.60–45.10)	0.14
	Illiterate	122	19.7	0.8	3.37 (1.50–7.59)	**0.003**	1.09 (0.07–17.63)	0.95
**Occupation**^a^	Other occupation	113	8.8	0.9	Reference			
	Farmer	179	15.6	1.1	1.91 (0.89–4.10)	0.10	1.27 (0.11–14.12)	0.85
	Unemployed	89	15.7	4.6	1.92 (0.81–4.56)	0.14	5.27 (0.58–48.02)	0.14
**Religion**^a^	None	19	26.3	0.0	Reference			
	Muslim	76	5.3	0.0	0.16 (0.04–0.65)	**0.01**	0.25 (0.00–13.26)	0.50
	Christian	210	13.3	1.0	0.43 (0.14–1.29)	0.13	0.47 (0.02–10.09)	0.63
	Traditionist	76	21.1	6.6	0.75 (0.23–2.38)	0.62	3.00 (0.16–56.64)	0.46
**Presence of domestic animals on compound**	Dogs Yes	300	26.3	4.3	1.11 (0.82–1.50)	0.52	1.23 (0.63–2.40)	0.55
	No	786	24.4	3.6	Reference			
	Cats Yes	260	26.5	3.5	1.12 (0.81–1.53)	0.50	0.89 (0.42–1.89)	0.76
	No	826	24.5	3.9	Reference			
	Goats Yes	633	28.3	3.9	1.55 (1.16–2.06)	**0.003**	1.12 (0.59–2.13)	0.72
	No	453	20.3	3.5	Reference			
	Cows Yes	339	28.3	3.8	1.29 (0.97–1.73)	0.08	1.02 (0.52–2.00)	0.94
	No	747	23.4	3.7	Reference			
	Chicken Yes	801	24.3	3.9	0.88 (0.65–1.20)	0.44	1.11 (0.54–2.29)	0.79
	No	285	26.7	3.5	Reference			
	Ducks Yes	215	25.6	4.7	1.04 (0.74–1.47)	0.81	1.32 (0.64–2.74)	0.45
	No	871	24.8	3.6	Reference			
**Access to health care**								
**Time to reach the nearest health facility (min)**	0–9	70	37.1	11.4	Reference			
	10–19	192	21.9	2.1	0.47 (0.26–0.86)	**0.01**	0.16 (0.05–0.57)	**0.004**
	20–29	184	28.8	7.6	0.68 (0.38–1.22)	0.20	0.64 (0.26–1.60)	0.33
	30–59	436	23.9	2.1	0.53 (0.31–0.90)	**0.02**	0.16 (0.06–0.44)	**0.001**
	≥60	204	22.5	2.9	0.49 (0.27–0.88)	**0.02**	0.23 (0.08–0.70)	**0.01**

^a^ calculated for individuals ≥18 years (n = 388)

The bivariate analyses identified many risk factors for tungiasis (Table 4). These included being of male sex (OR = 1.59, p = 0.001) and age < 15 and ≥ 40 years (OR between 4.04 and 12.45, p<0.001 and p<0.01, respectively). Living in a house with a floor of sand/earth (OR = 4.31, p < 0.0001) and mud walls (OR = 4.11, p < 0.0001) were significantly related to the occurrence of tungiasis. Other significant risk factors were: using a traditional latrine or bush as a toilet; spreading waste on the compound or disposing waste on a pile; using mud puddles as a water source (all p < 0.05); a low frequency of washing (only once a day, OR = 1.99, p<0.0001) and not using soap (OR = 3.81, p<0.001); living in crowded houses (4–6 persons per household, OR = 1.69, p < 0.05); sleeping together with many other persons in a room (p < 0.001) or children sleeping on the floor (OR = 1.89, p < 0.001). In individuals 18 years or older, not completing primary school or never having attended primary school at all increased the odds of being affected by tungiasis by a factor of three (OR = 3.37, p<0.05, Table 5).

On conducting the multivariate analyses, only the demographic exposure variables male sex and age under 15 remained highly significant (Table 6). Exposure variables indicating a low economic status such as poor construction characteristics of the house, direct sleeping on the floor, many people sleeping in a single room and restricted access to water also remained as significant factors.

**Table 6:** Risk factors of tungiasis/severe tungiasis after multivariate analysis.

	Presence of tungiasis		Presence of severe tungiasis (> 30 lesions)	
	Adjusted OR (95% CI)	P value	Adjusted OR (95% CI)	P value
Being of male sex			2.29 (1.18–4.46)	0.01
Age				
0–4	8.90 (2.94–26.89)	<0.0001		
5–9	12.88 (4.31–38.54)	<0.0001		
10–14	7.23 (2.37–22.02)	<0.0001		
40–59	3.49 (1.07–11.39)	0.04		
≥ 60	5.32 (1.50–18.85)	0.01		
Using mud puddles as water source			25.48 (3.50–185.67)	0.001
Washing only once a day			2.23 (1.11–4.51)	0.03
Using soap when washing:				
sometimes	1.57 (1.09–2.28)	0.02		
never	7.36 (3.08–17.62)	<0.0001		
Staying in a house with:				
4.5–6 persons/sleeping room	1.77 (1.07–2.93)	0.03		
children sleeping on the floor	1.68 (1.03–2.74)	0.04		
Time to health facility 10–19 min	0.47 (0.23–0.95)	0.04	0.20 (0.06–0.69)	0.01
30–59 min			0.12 (0.04–0.34)	<0.0001
≥ 60 min			0.22 (0.07–0.68)	0.009
Living in a house with mud walls	3.35 (1.71–6.58)	<0.0001		

Population Attributable Fractions were calculated for those variables which are amenable to modification (Table 7). The PAF for living in a house with mud walls was 64.45%, for washing without soap 16.61% and washing only once a day 20.18%.

**Table 7:** Population attributable fractions for exposure variables amenable to modification.

	AR (%)	% exposed among cases	PAF (%)
Washing only once a day	55.16	36.6	20.18
Not using soap when washing	36.31	45.8	16.61
Staying in a house with mud walls	70.15	91.9	64.45

PAF is the fraction of cases which would not have occurred if an exposure had been avoided and is calculated as % exposed among cases x attributable risk (AR).

AR is the risk of tungiasis in the exposed group due to the exposure and is calculated as (OR– 1)/OR.

## Discussion

Tungiasis is a NTD prevalent in resource-poor communities in South America, the Caribbean and sub-Saharan Africa [3–7]. Although the disease is associated with important morbidity, it is neglected by health care providers globally [2,20–23]. Widespread control has never been attempted, only isolated efforts to treat infected individuals, often by non-governmental organizations. In East Africa, this is largely due to the lack of data on prevalence and severity of disease and hitherto risk factors have only been investigated in restricted age groups.

This study showed a prevalence of 25% in the overall study population and 33.8% in children under 15 years. The overall prevalence is similar to that found in a community-based study in Central Uganda (where the median prevalence in humans was 22%, but only animal keeping households were included), but considerably lower than prevalences observed in rural and urban resource-poor communities in Brazil and Nigeria (with prevalences up to 45%) [6,7,20,24,25]. Age-specific prevalence followed an S-shape curve, peaking in the 5 to 9 year age group and the elderly, an unusual epidemiological characteristic which seems to be true for all geographic areas and independent of the overall prevalence [6,7,15,21]. This may be due to certain age-specific behavioural patterns associated with different degrees of exposure, e.g. young children playing on the ground, as suggested by Muehlen et al. [6] and the elderly spending large amounts of time lying on the ground. Other hypotheses are a protecting effect of the increasing corneal layer of the feet [26,27], a higher level of practice and dexterity in taking out embedded sandfleas with increasing age [7] and more attention given to personal hygiene.

More than half of all cases (52.8%) had a low intensity of infection (less than 6 lesions), while 15% had more than 30 lesions. The percentage of patients with severe tungiasis was lower than observed in Brazil [7,15,20,24,25]. However, this is not surprising, taking into account that prevalence and intensity of infection are positively correlated [6,21,28]. The observation that age-specific prevalence significantly correlated to high intensity of infection (rho = 0.90; Fig 3) confirms that children and the elderly bear the highest burden of disease. Anecdotal reports show that elderly individuals without social support structures tend to be infected with tungiasis more frequently [21]. This tendency was confirmed in this study, although it was not significant.

Tungiasis is a zoonosis in which sylvatic, peri-domiciliary and domestic cycles are interlinked in a complex manner [2]. The situation becomes even more intricate when transmission also occurs inside the house, without the involvement of an animal reservoir. Intra-domiciliary transmission indicates that the off-host cycle of *T*. *penetrans* is completed inside the house. Usually, this is a room in which family members spend many hours a day, such as the sleeping room. If the floor in this room consists of sand, dried mud or rugged cement with holes and cracks, eggs that have been expelled by embedded female sand fleas overnight and which have fallen on the floor are swept into crevices of the floor or into the cracks between floor and wall, when the room is cleaned with a broom in the morning. Eggs can develop into larvae and pupae in such cracks [29].

That intra-domiciliary transmission occurs in the study area is supported by the finding that direct sleeping on the floor or if walls of the sleeping room consisted of mud remained significant risk factors in the multivariate analysis. The more people slept in a room the higher were the odds of tungiasis in household members.

It is known that different animal species act as reservoirs in different countries [25,30,31]. In our study population, 74% of all households had chicken, 60% had goats, 25% had dogs and 25% had cats. However, no specific animal species was identified as a risk factor for tungiasis in this study. This finding supports the assumption that perhaps in these coastal communities the *Tunga penetrans* cycle is almost entirely human and does not involve animal reservoirs. It should be noted that animals were not examined for infection in this study, only observed as present in the compound and reported as to where they sleep at night (S3 Appendix). In Northeast Brazil, stray dogs and cats are important reservoirs in urban areas, whereas in rural areas pigs are the most import species [30,31]. Pigs were also identified as the major reservoir *of T*. *penetrans* in Nigeria and in Uganda [15,25]. However, pigs were not kept in any of the households in the study area, because a considerable part of the population is Muslim. Actually, being Muslim was identified as a significant protective factor in the bivariate analysis (Table 5), which may be explained by the fact that Muslims wash their feet several times a day before entering the mosque for prayer.

Other risk factors which remained significant after multivariate regression analysis were the limited access to water (water only available from muddy pools), frequency of washing as well as bathing without soap. A similar finding was made in a resource-poor community in Northeast Brazil [14]. It is tempting to speculate that these risk factors are correlated to the reproductive biology of *T*. *penetrans*. Female sand fleas are fertilized by males exploring the skin only after females are embedded in the epidermis and have started neosomy [32]. There is circumstantial evidence that males are attracted by odor emitted from the faecal material released by females in regular intervals [12,13]. The faecal material spreads into dermal papillae around the lesion, and since it is very sticky, it needs soap to be washed off. Hence, when soap is not used or unavailability of water prevents any washing at all, more male sand fleas should be attracted to the skin and, hence, more females will be fertilized. Over time, this will lead to a higher intensity of infection.

It has previously been reported that within endemic areas, tungiasis is heterogeneously distributed [2]. This was confirmed in this study: where prevalence varied between villages from 7.8% to 64.7% in the five study villages in Malanga Sub-location, all situated within 4 km of each other and from 13.4% to 35.5% in the three study villages in Kakuyuni Sub-location, within 2 km of each other. Whether the heterogeneity is determined by differences in the predominant type of exposure within a community, such as intra-domiciliary versus peri-domestic could not be clarified in this study.

We found very high Population Attributable Fractions for biologically very plausible variables. Trickling of sand and dust from mud walls creates ideal conditions for the off-host life cycle of sand fleas in cracks of the floor. Building walls of stone or cement would reduce the prevalence of tungiasis by 64 percent. Similar, promoting better hygiene, particularly washing with soap, would reduce the prevalence of tungiasis in the community by 17 and 20%, respectively.

We realize that this study has several limitations. First, there is an overrepresentation of adult females in the study group. The study was conducted during the day on all days of the week, including Saturday and Sunday, in order to encounter school children on the compound. However, since the majority of adult males in our study population worked as farmers and returned only after sunset we could not examine them. Extending our working periods towards the evening was not possible due to insufficient lighting and safety concerns. The distances between the households in Malanga and our time constraints, also meant that there were fewer households included in the study from this area than from Kakuyuni. Ecologically the two areas are quite different.

Taken together, many factors which—by one way or another—are linked to poverty were identified as important risk factors in the bivariate and/or multivariate regression analysis, such as poor construction characteristics of the house, absence of a ventilated pit latrine, no access to drinking water on the compound, a single sleeping room for children and adults, absence of beds and mattresses, unavailability of soap for body wash, an asset score below 5 points and a low level of education among adults. Thus, as seen elsewhere in the world, tungiasis in rural Kenya is a poverty-associated disease in which the poorest of the poor bear the highest burden of disease, but that it can be controlled with simple housing improvements, improved access to water and hygiene practices.

## Supporting information

S1 Appendix Household informed consent. (DOCX)

S2 Appendix Database. Prevalence of tungiasis in 5 schools in Kilifi County.
(DOCX)

S3 Appendix Domestic husbandry practices. (DOCX)

S4 Appendix STROBE checklist. (DOC)
