# Supplementary material for: Prevalence, intensity and risk factors of tungiasis in Kilifi County, Kenya: I. Results from a community-based study
Source: PLoS Negl Trop Dis. 2017 Oct 9;11(10):e0005925. doi: 10.1371/journal.pntd.0005925 (PMC5648262; doi:10.1371/journal.pntd.0005925)
Supplement: S1 Appendix — (DOCX) [file pntd.0005925.s001.docx]

**Household Informed Consent**

The Ministry of Health in Kenya would like to control jiggers in all areas of the country. To start with they must find out who gets jiggers, why and where. With this information they can then design the best way to fight jiggers and stop the suffering.

In order to do this, the Ministry of Health in Kilifi County, Dabaso Community Unit, is working with the University of Berlin to conduct the necessary surveys in schools and in homesteads. Your household has been selected to participate in the study. We would like to ask you some questions about yourself, your family members and about your homestead. We will be observing things in your compound such as whether you have animals and what your house and the floor are made of. We would also like to look carefully at the feet of all members of the family to observe whether they have jiggers, and how many they have.

The study team visiting you today includes (give name of interviewers). They are members of Dabaso Community Unit, Public Health Officers and a visiting PhD student from Germany, Susanne Wiese.

The study will not be taking any samples from you or your family members. We will only ask questions and make observations. The information we collect from you will be entered into a computer together with that from all of the houses that we visit. Someone with special training will analyse the information. No one from your community will ever see your answers to the questions. The forms will be kept in a locked office away from your community. It is important that we have your names so that the treatment team can come back to help you with the jiggers in future.

While we will not share individual information, we will come back to tell you about what we found for all of the households together, and how we will use that to plan the jigger control. We will also share these findings with other organisations and to the Kilifi County and national health management teams.

When we have finished asking questions another group will treat anyone who has jiggers and they will return to provide treatment on another day and make sure that the jiggers are all dead. If anyone is found to have any other disease we will advise you on the best way to manage it. No one will be given anything for participating in the study, except treatment of their jiggers if they have them.

You do not have to participate in this study. It is only voluntary. If you tell us now that you do not want us to ask you the questions we will leave and go to the next house. If you are happy to join the study please sign the form as shown by the interviewer.

**Consent to Take Part in Research**

I freely give my consent to take part in this study and authorize that my information as described above and that of all members of my household, be collected in this study. I understand that by signing this form I am agreeing to take part in research.

_____________________________________________ ____________

Signature of Person Taking Part in Study Date

_____________________________________________

Printed Name of Person Taking Part in Study

**Statement of Person Obtaining Informed Consent**

I have carefully explained to the person taking part in the study what he or she can expect from their participation. I hereby certify that when this person signs this form, to the best of my knowledge, he/ she understands:

- What the study is about;
- What procedures will be used;
- What the potential benefits might be; and
- What the known risks might be.

I can confirm that this research subject speaks the language that was used to explain this research and is receiving an informed consent form in the appropriate language. This person is able to hear and understand when the form is read to him or her. This subject does not have a medical/psychological problem that would compromise comprehension and can, therefore, give legally effective informed consent.

In addition, I confirm that all of the elements of informed consent have been presented to the subject according to the summary presented to the Pwani University IRB for review and approval.

_________________________________________________ ____________

Signature of Person Obtaining Informed Consent Date

__________________________________________________

Printed Name of Person Obtaining Informed Consent
