# Supplementary material for: Prevalence, intensity and risk factors of tungiasis in Kilifi County, Kenya: I. Results from a community-based study
Source: PLoS Negl Trop Dis. 2017 Oct 9;11(10):e0005925. doi: 10.1371/journal.pntd.0005925 (PMC5648262; doi:10.1371/journal.pntd.0005925)
Supplement: S2 Appendix — Prevalence of tungiasis in 5 schools in Kilifi County. (DOCX) [file pntd.0005925.s002.docx]

http://dx.doi.org/10.7910/DVN/GI1BM5
