# Supplementary material for: Prevalence, intensity and risk factors of tungiasis in Kilifi County, Kenya: I. Results from a community-based study
Source: PLoS Negl Trop Dis. 2017 Oct 9;11(10):e0005925. doi: 10.1371/journal.pntd.0005925 (PMC5648262; doi:10.1371/journal.pntd.0005925)
Supplement: S3 Appendix — (DOCX) [file pntd.0005925.s003.docx]

|  | | Number of households with domestic animals (%), N = 233 | | | | | |
| --- | --- | --- | --- | --- | --- | --- | --- |
|  |  | Dogs | Cats | Goats | Cows | Chicken | Ducks |
| Household ows animal species |  | 59 (25.3) | 59 (25.3) | 140 (60.1) | 70 (30.0) | 172 (73.8) | 42 (18.0) |
| Animals are present on compound during visit. | Loose | 43 (18.5) | 46 (19.7) | 16 (6.9) | 16 (6.9) | 155 (66.5) | 34 (14.6) |
|  | Tied | 4 (1.7) | 0 (0.0) | 58 (24.9) | 20 (8.5) | 1 (0.4) | 1 (0.4) |
|  | Caged | 2 (0.9) | 0 (0.0) | 7 (3.0) | 0 (0.0) | 1 (0.4) | 0 (0.0) |
| Animals stay inside the house during the night. |  | 3 (1.3) | 42 (18.0) | 30 (12.9) | 1 (0.4) | 78 (33.5) | 18 (7.7) |
| Animals stay on the compound during the night. |  | 58 (24.9) | 39 (16.7) | 98 (42.1) | 61 (26.2) | 108 (46.4) | 23 (9.9) |
